# Characterization of dielectric properties of residual ashes obtained from rice husk and annoni grass

**DOI:** 10.1038/s41598-023-40887-y

**Published:** 2023-09-02

**Authors:** Vinícius Macedo Pereira, Marcos V. Thomas Heckler, Marcos A. Z. Vasconcellos, Eliana W. de Menezes, Luis E. G. Armas

**Affiliations:** 1 Grupo de Óptica, Micro e Nanofabricação de Dispositivos-GOMNDI, Universidade Federal do Pampa, UNIPAMPA, Campus Alegrete, Alegrete, CEP 97546-550 Brazil; 2 Laboratório de Eletromagnetismo, Micro-Ondas e Antenas, Universidade Federal do Pampa-UNIPAMPA, Campus Alegrete, Alegrete, CEP 97546-550 Brazil; 3 Instituto de Física, UFRGS, CP 15051, Porto Alegre, RS CEP 91501-970 Brazil; 4 Laboratório de Sólidos e Superfícies, Instituto de Química, Universidade Federal do Rio Grande do Sul-UFRGS, CP 15051, Porto Alegre, RS 91501-970 Brazil

**Keywords:** Environmental sciences, Engineering, Materials science

## Abstract

This paper presents a study about the characterization of dielectric properties (dielectric constant ${\varepsilon }_{r}$ and dielectric-loss constant $\mathrm{tan}\delta$) of samples derived from two different biomass types: rice husk ash (RHA) and annoni grass ash (AGA).The procedure is carried out using the resonant cavity method along with a vector network analyzer. For this purpose, four different ashes were produced, by burning rice husk and annoni grass at two different temperatures and burning times: 400 °C/30 min (RHA40030 and AGA40030) and 800 °C/5 h (RHA8005h and AGA8005h). These ashes were combined with Bakelite to produce cylindrical samples with diameter of 30 mm and thickness of 4.5 mm, which were characterized considering the frequency in the test band for 5G technologies (${f}_{o}=3.5$ GHz). Experimental results showed that the samples burnt at high temperature showed very high $\mathrm{tan \delta }$ when compared to the samples burnt at low temperature, mainly for AGA8005h. These values are $\mathrm{tan \delta }=$ 0.1690 and 1.4900 for RHA8005h and AGA8005h, respectively. The resulting dielectric constants are ${\varepsilon }_{r}$ = 3.87 for RHA8005h and ${\varepsilon }_{r}$ = 15.14 for AGA8005h). These very high dielectric loss tangent, indicate that these materials exhibit formidable properties for electromagnetic energy absorption, hence allowing the application as radiation-absorbent material (RAM).

## Introduction

In times of technology and communication, the frequency in microwave range is used in various sectors such as, military applications, technology, radar, wireless communication, satellite^1^. Nowadays, humans are habitual of laptops, mobile phones, Bluetooth devices, Wi-Fi networks that inadvertently get exposed to radiofrequency radiation, producing electromagnetic (EM) pollution in the environment^2^. This EM pollution pollutes the environment and causes many health issues to human like depression, sleep disorders, headache^2^ etc., resulting hazardous for humans, so it becomes necessary to control EM pollution. EM pollution can be curbed by the use of absorbing materials or shielding techniques. The absorbing approach is the best substitute for developing microwave absorption materials (MAMs), which helps to eliminate EM interference.

Nowadays, agriculture wastes are being used as an alternative materials to absorb microwave interference in different applications such as those mentioned before. In this sense, in Southern Brazil, rice cultivation is one of most important agricultural activities,

in terms of both cultivation area and productivity, which results in a generation of great quantities of agricultural residues that do not have added commercial value. Particularly, the resultant rice husk ash (RHA) of the rice husk burning process is a material, which at the present time is being used as EM wave absorber. For instance, Seng et al.^3^ have reported a new approach for fabricating an EM wave (microwave) absorber with low density and strong absorption by using RHA and carbon nanotubes (CNT) composites. They investigated the performance of RHA and CNT composites operating as a stacking multiple-layered microwave absorber. The fabricated multilayered absorbers behaved like a multiple narrow-bands microwave absorber, with a low reflectivity (< − 15 dB) over the frequency range of 2–18 GHz.

Seng et al.^4^ proposed the development of two different electromagnetic absorbing materials: the first, based in the mixture of RHA and Polyethylene (PE) resin; and the second, based in this mixture (RHA and PE resin) with addition of 2wt% of carbon nanotubes, increasing the electromagnetic absorption in ~ 90%. Garnayak et al.^5^ developed a study about other types of potential electromagnetic absorber materials considering samples produced from China clay and RHA. These materials were characterized in the X-band for the suppression of radio interference in radar and communication systems.

On the other hand, annoni grass (*Eragrostis plana Nees*) is a perennial grass species commonly found in South Africa and was accidentally introduced in Brazil in mid 1950’s. In State Rio Grande do Sul, Brazil, annoni grass (AG) was cultivated as forage pasture for livestock. However, it was found that it is unsuitable for this use due to its low nutritional quality, thus resulting in production losses. Therefore, AG is seen as an invasive pest in nature, which also competes with native pasture for nutritional resources in the soil^6^, being of great interest to develop sustainable alternatives for its final removal from the Brazilian territory. For this purpose, it is necessary to develop a research for a possible use of annoni grass ash (AGA) as EM wave absorber.

In this sense, the study of the relative electric permittivity ${\varepsilon }_{r}$ (also known as dielectric constant) and the dissipation factor $\mathrm{tan}\delta$ (also known as loss tangent) of RHA and AGA and its applicability is of great importance for the sustainable reuse of the agricultural wastes of rice cultivation and AG. For instance, Liu et al.^3^ reported a study about the characterization of dielectric properties of different types of RHA produced with five burning processes with temperatures varying in the range of 400–800 °C during 2 h. The experimental analysis were performed considering the frequency range of 2–10 GHz, and the RHA was deposited on a paraffin substrate for its insertion into the measuring device (a coaxial transmission line).

Therefore, in order to look for possible applications to RHA and AGA as EM absorbers, this paper presents the characterization of dielectric constant and dielectric-loss constant of samples produced from three different combinations of RHA and AGA with bakelite (agglutinative agent) concentrations using the resonant cavity method. Two different burning conditions were considered to produce the ashes: 400 °C/30 min and 800 °C/5 h. The produced samples have been characterized by Raman spectroscopy, X-ray Diffraction (XRD), Scanning Electron Microscopy (SEM), Energy dispersive X-ray spectroscopy (EDS) and X-ray Fluorescence (XRF).

## Experimental

### Design and specifications of the experimental setup

**Figure 1:**
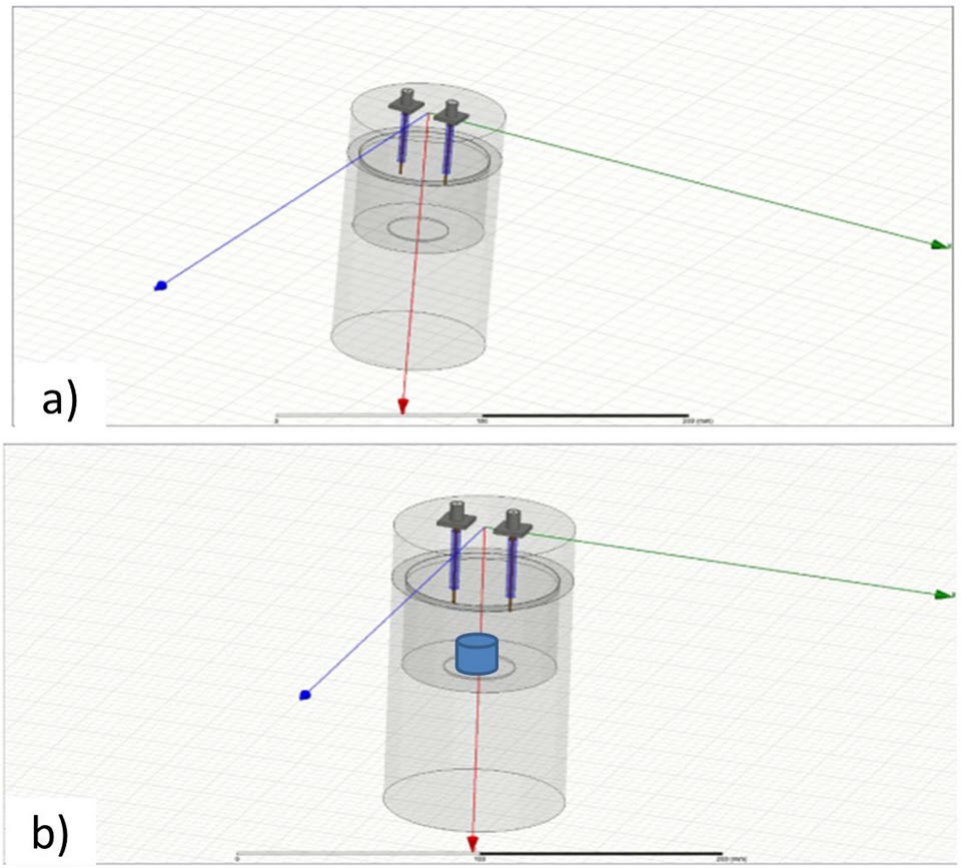
Simulation model of the resonant cavity in ANSYS HFSS software: (**a**) empty cavity (EC) and (**b**) loaded cavity with cylindrical sample.

The cylindrical resonant cavity used in this study, for the characterization of dielectric properties, has been fabricated by machining a solid piece of aluminum, which is costly effective and exhibits high conductivity in the microwave frequency range. The electromagnetic field inside the cavity can be excited by means of two probes, which are positioned on the top face symmetrically off the center (Fig. 1a), hence allowing measuring power transmission between the ports.

The central frequency of this analysis was defined to be 3.5 GHz, which is in one of the frequency bands allocated for testing new materials and technologies for the 5th generation of mobile telecommunications system (5G)^8–14^. The physical dimensions of this prototype have been optimized using the electromagnetic simulator ANSYS HFSS software. The simulation model used in this optimization is shown in Fig. 1a, where the empty cavity (i.e. without any sample of the material under test) should resonate at . After the optimization process, the final internal dimensions are: internal radius *a* = 32.25 mm; internal height *d* = 41.50 mm and length of each excitation probes (copper wires) *l* = 7.56 mm. On the other hand, the modeling of this device loaded with the cylindrical sample is shown in Fig. 1b, where it is indicated that the samples should be positioned at the center of the opposite face from the excitation elements.

**Figure 2:**
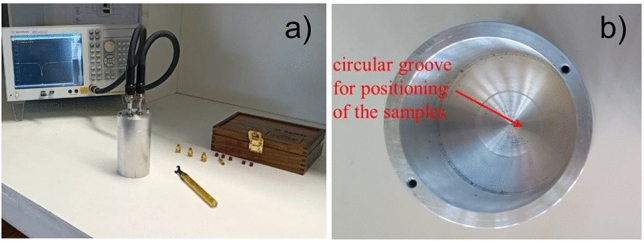
(**a**) Experimental setup for the characterization of the fabricated samples, showing the resonant cavity and a vector network analyser; (**b**) circular groove in the internal region of resonant cavity.

The experimental setup for characterization of dielectric properties is shown in Fig. 2a and is composed of the resonant cavity, where the samples are inserted in, and a vector network analyzer model E5071C—Agilent Technologies, which is used for the measurements of S-parameters. In order to ensure that the samples are positioned centrally inside the resonant cavity, a small circular groove with diameter of 30 mm and depth of 1 mm was etched in the bottom wall of the cavity, as highlighted in Fig. 2b.

As stated above and shown in Fig. 1a, the cavity has two probes that allow exciting the electromagnetic field inside the cavity. The measurement is carried out so that both reflection (*S*_11_) and transmission (*S*_21_) coefficients are obtained. The parameter *S*_11_ is determined by coupling power into the cavity at one port and measuring the power reflected back to the analyzer from the same port; hence *S*_11_ allows estimating the reflected power and can be used to determine the resonance frequency of the loaded cylindrical cavity. Due to the geometrical symmetry, the reflection coefficient is nearly independent on the choice of the port (*S*_22_ ≈ *S*_11_). The parameter *S*_21_ is obtained by coupling power into the cavity at one port and measuring the power transmitted to the analyzer through the other port; hence *S*_21_ allows determining the losses that occur inside the cavity. Hereby one can point out that the losses are mainly due to the material under test, since aluminum performs as a very good conductor at the testing frequency.

**Figure 3:**
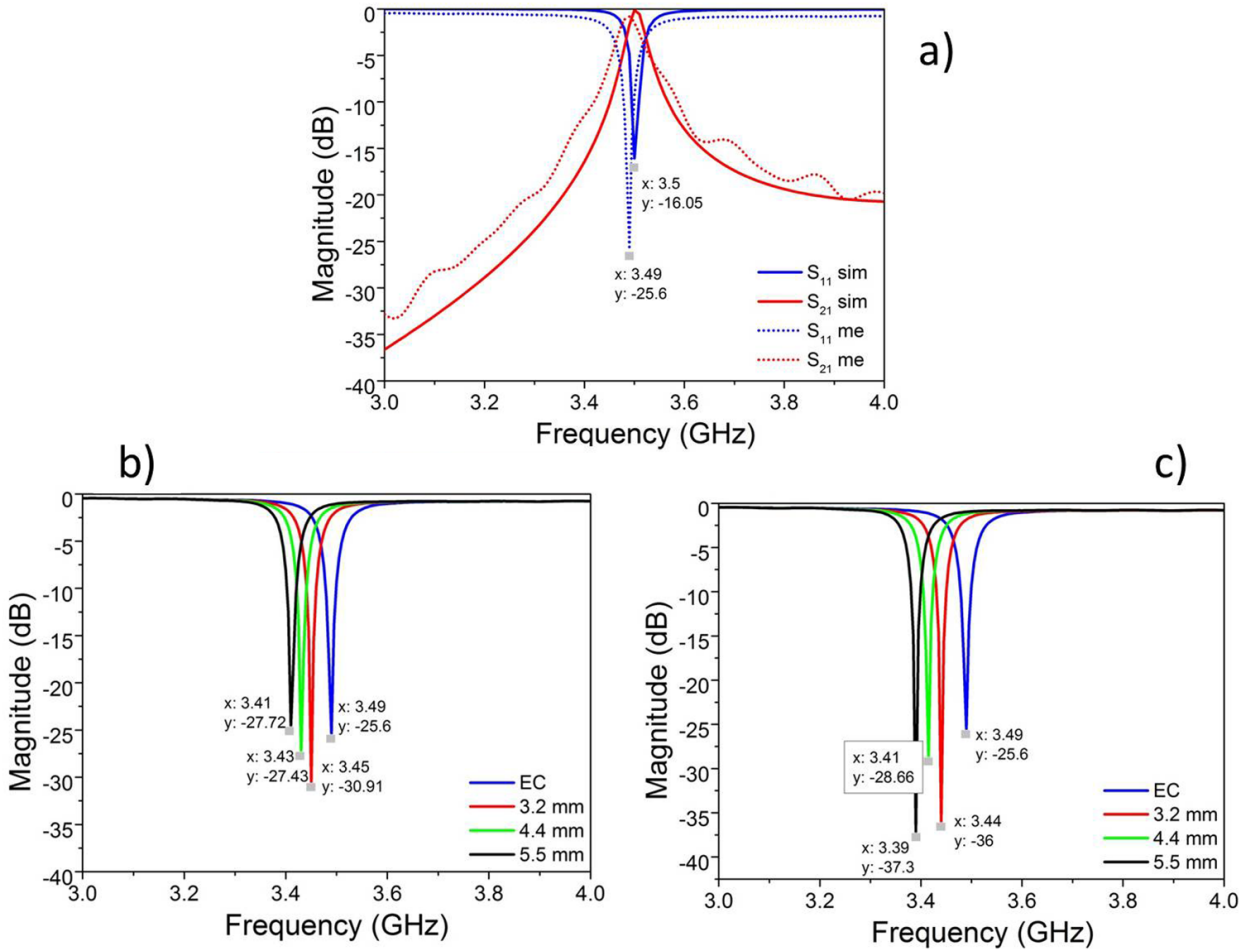
(**a**) Comparison between the simulated and measured *S*_*11*_ and *S*_*21*_ parameters in EC; (**b**) measured *S*_*11*_ parameters of PEAD samples; (**c**) measured *S*_*11*_ parameters of Nylon samples.

Figure 3a shows the simulated and measured *S*_11_ and *S*_21_ parameters with the empty cavity (EC). Where it is possible to see that the cavity resonates at ${f}_{0}=3.4$9 GHz, which is slightly lower than the considered central frequency (3.5 GHz). This small difference can be attributed to the intrinsic precision during the construction process of the cylindrical cavity. However, the resonance measured is satisfactorily close to the initial central frequency of the project (3.5 GHz).

For practical measurements, the cavity was first calibrated with dimensionally identical samples of known permittivity, such as ${\varepsilon }_{r}=3.10$ for Nylon and ${\varepsilon }_{r}=2.25$ for PEAD. For this purpose, four samples of both Nylon and PEAD with a diameter of 28 mm and different thickness ($h$) ($h$ = 3.2, 4.4 and 5.5 mm) were prepared. Before the measurements, simulations of the variation of resonant frequency of the loaded cavity (${f}_{a}$) with the samples of different thicknesses and materials were performed. For each combination of thickness and material, four samples were observed with ${\varepsilon }_{r}$ varying within their typical tolerance level. In these simulations, the concept of analysis of variance (ANOVA)^15^ was applied. From this analysis, it was verified that the variation of ${f}_{a}$, and therefore ${\varepsilon }_{r}$, is dependent of the thickness and material type^16^ (see Fig. 5 on Ref.^16^). Because, different thicknesses imply different volumes of dielectric material inside the cavity, producing a down shift of ${f}_{a}$, respecting to ${f}_{o}$. From this shift was also verified that using samples with a thickness of 4.4 or 5.5 mm, the variation of ${f}_{a}$, considering different materials, is more visible and, consequently, it would allow greater precision for ${\varepsilon }_{r}$ calculation. For this reason, the samples in this work were fabricated with a thickness of 4.5 mm.

In order to validate, the results obtained by the simulations, the *S*_*11*_ parameters for PEAD and Nylon samples were measured, as shown in Fig. 3b,c. These figures show that as the thicknesses increase (3.2–5.5 mm) the resonance frequencies are shifted to lower values, respecting to the resonance frequency for EC. The measured resonance frequencies for each sample are approximately similar to the average frequencies observed in simulation^16^ (see Fig. 5 on Ref.^16^). Table IV on Ref.^16^ shows a comparison between the simulated and measured resonance frequencies for PEAD and Nylon samples with different thickness, where it is possible to see a difference of 10 MHz between the simulated and measured results. The same difference was also observed in simulated and measured results for empty cavity, as shown on Fig. 3a

For determination of $${\varepsilon }_{r}$$, a mathematical modeling has been developed between the ratio ($${f}_{0}-{f}_{a})/{f}_{0}$$ and $${\varepsilon }_{r}$$ to be calculated, taking as base, the study proposed by Rubinger and Costa^17^ and adjusting the formalism for cavity geometry and samples of this study. The simulation model in HFSS® was modified, so that $${f}_{0}=3.4$$9 GHz and, subsequently, electromagnetic simulations were carried out varying the $${\varepsilon }_{r}$$ from 2 to 20 with a step of 1 for each of the three samples with different thicknesses analyzed. The obtained results were used to plot the three calibration curves, shown on Fig. 10 of Ref^16^. It is observed that, unlike Rubinger and Costa^17^, where their experiment has been described mathematically by means of a linear relationship, the present experiment, in turn, is satisfactorily modelled mathematically through logarithmic equations. Using Excel® software, the equations that model each curve are shown in Fig. 10 of Ref.^16^. For a simple illustration, Eq. (1) shows the mathematical model for $$h$$ = 4.4 mm. It is worth to emphasize that the equations will be different for each sample with different thickness, diameter and so on.

1 $$\frac{{f}_{0}-{f}_{a}}{{f}_{0}}=0.0094\mathrm{ln}\left({\varepsilon }_{r}-1\right)+0.0145.$$

For the ${\varepsilon }_{r}$ calculation of each sample (according to its thickness), Eq. (1) was manipulated, in order to leave the term ${\varepsilon }_{r}$ on the left side of the equality. As a result, Table 1 shows the results of ${\varepsilon }_{r}$, calculated for each measured sample. These results show considerable reliability to those commercially specified. For PEAD is observed a small variation between the values calculated for each sample of different thickness. This variation can be considered within the tolerance limits (2.3–2.45) that are usually specified by the manufacturers and several authors^18^. However, for the Nylon samples, the calculated dielectric constant ${\varepsilon }_{r}$ resulted in values substantially close to 3.1, which is its commercially specified value and reported on the literature (3.09–3.21)^18^. Therefore, according to these results, we can see that it is possible to use the designed and fabricated resonant cavity for dielectric properties characterization of different materials.

**Table 1:** Resonance frequencies ${f}_{a}$ and dielectric constant ${\varepsilon }_{r}$ for Pead and nylon samples with different thicknesses.

$h$ (mm)	PEAD		Nylon	
	${f}_{a}(GHz)$	${\varepsilon }_{r}$	${f}_{a}(GHz)$	${\varepsilon }_{r}$
3.2	3.45	2.23	3.44	3.16
4.5	3.43	2.33	3.41	3.10
5.5	3.41	2.43	3.39	3.06

Following the same procedure described before, the mathematical modelling, for the experimental setup in this work, was derived from a set of simulations carried out with the electromagnetic simulator ANSYS HFSS. In the simulations, the constant ${\varepsilon }_{r}$ of the samples was also varied in the range from 2 to 20. The resultant values of resonant frequency of the loaded cavity ${f}_{a}$ were applied, and the following equation was obtained:

2 $$\frac{{f}_{0}-{f}_{a}}{{f}_{0}}=0.0108\mathrm{ln}\left({\varepsilon }_{r}-1\right)+0.0158$$

By isolating ${\varepsilon }_{r}$ in Eq. (2), it comes out that:

3 $${\varepsilon }_{r}={e}^{[\frac{\frac{({f}_{0}-{f}_{a)}}{{f}_{0}}-0.0158}{0.0108}]}+1$$

Equation (3) is valid only for the mathematical representation of the proposed experiment, considering the geometries of the cavity and samples and for ${f}_{0}=$ 3.49 GHz. The determination of dielectric-loss tangent $\mathrm{tan \delta }$ was done by a set of parametric simulations in ANSYS HFSS. The procedure is simply done by performing simulations, each for a different value for the loss tangent of the sample in the electromagnetic model. The loss tangent corresponding to the material under test is the one that produces the *S*_21_ curve that best fits the measured data, particularly the maximum level of this parameter and the frequency ${f}_{a}$ in which it occurs.

### Synthesis of RHA and AGA

Initially, the precursor biomasses (rice husk and seared anonni grass) were properly cleaned by means of two washing processes: the first with potable water and the second with distilled water twice. Then, the RH and the seared anonni grass were placed in a stove for 24 h and 100 °C for complete drying.

**Figure 4:**
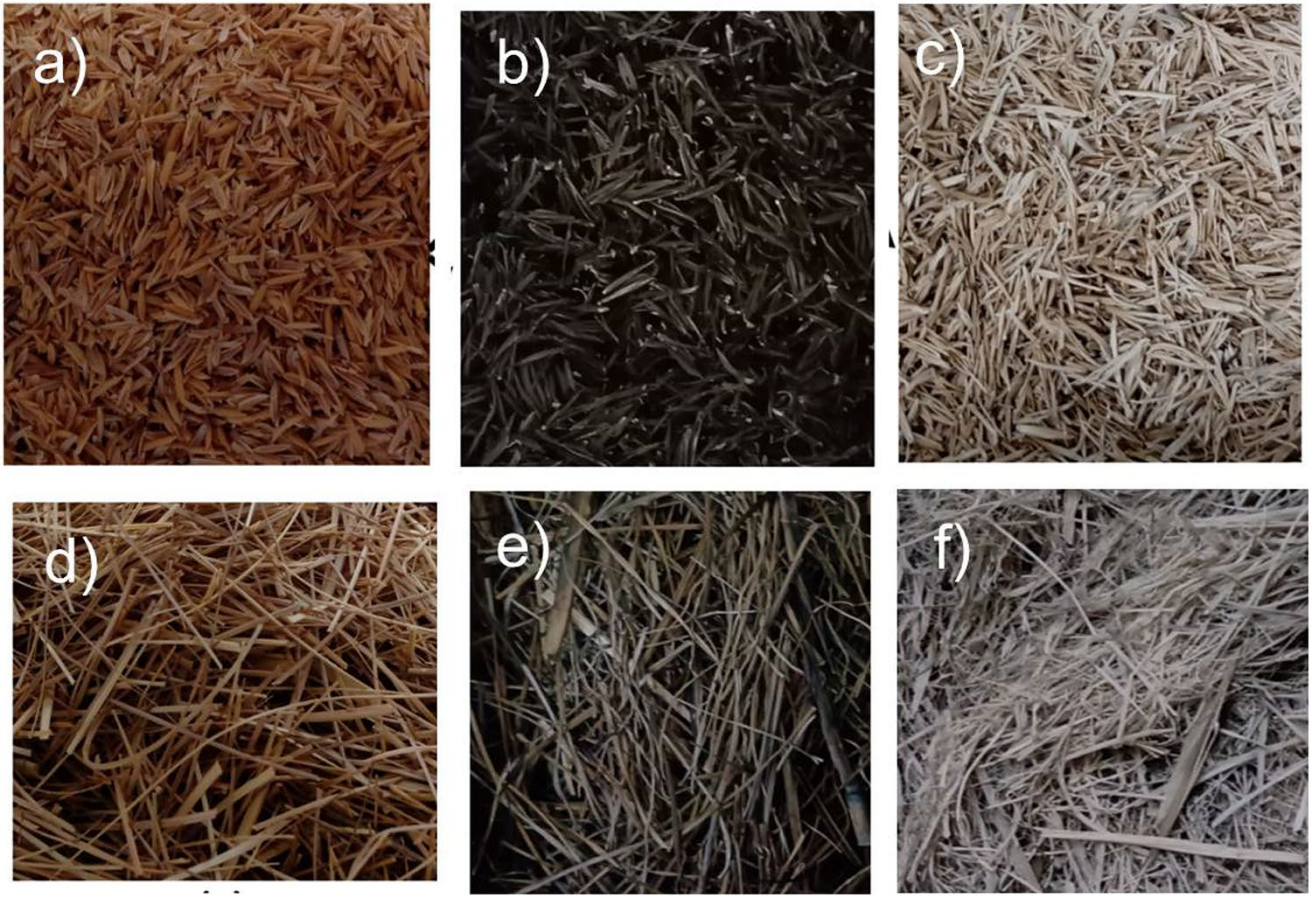
Rice husk (**a**) and annoni grass (**d**) used in this study prior to the burning process. (**b**) RH burnt with 400 °C/30 min (RHA40030); (**c**) RH burnt with 800 °C/5 h (RHA8005h); (**e**) AG burnt with 400 °C/30 min (AGA40030); (**f**) AG burnt with 800 °C/5 h (AGA8005h).

Figure 4a–f shows the RH (a), RHA (b,c), AG (d) and AGA (e,f) used in this work. For RHA and AGA production the RH and the AG were burnt in a muffle oven with a heating ramp of 10 °C/min, considering two different burning processes: by using 400 °C/30 min as suggested by Perez^19^ and Dalosto et al.^20^, the produced ashes exhibit black colour, high carbon concentration and lower density. Gonçalves^21^ and Lima^22^ suggested the use of 800 °C/5 h to yield ashes with gray colour (almost white) and predominant concentration of silica (specifically in the case of RHA).

Figure 4b shows the RHA produced at 400 °C/30 min (RHA40030), where it is possible to see its black color and low density, which is in accordance to the results presented by Perez and Dalosto et al. In Fig. 4c, the RHA produced at 800 °C/5 h (RHA8005h) is presented. This ash has gray color, and density similar to the one observed for the RHA40030 sample. These visual characteristics are according to the descriptions reported by Gonçalves^21^ and Lima^22^.

Figure 4d shows the AG used in this work. By using the same burning processes described above to produce ashes with AG, the resulting samples are now named as AGA40030 for 400 °C/30 min, shown in Fig. 4e, and AGA8005h for 800 °C/5 h, shown in Fig. 4f. The AGA40030 sample presents the same physical characteristics (black color and low density) reported by Dalosto et al. On the other hand, this ash yielded a voluminous material with lower density than RHA40030. The AGA8005h sample exhibits gray color similar to RHA8005h, but higher density than AGA40030 and RHA40030.

These four different ashes were obtained from 50 g of each biomass. Thereby, the different burning conditions resulted in different amounts (g) of ashes. From this information, the percentage yield *R* of each process in relation to the raw material was estimated. The masses (in grams) and the percentage *R* of each obtained ash were compared with the mass of raw material, according to the burning process. The results of this comparison are shown in Table 2. The ashes obtained from RH presented higher yield than ashes obtained from AG for the two burning conditions. This result was observed, because RH has smaller volume and is significantly heaviest than AG, resulting in ashes with higher density. Additionally, ashes obtained by the process using 800 °C/5 h presented *R* lower than the ashes obtained with 400 °C/30 min because, according to Liou^23^ and Onojah et al.^24^, at higher temperatures, reduction of organic matter occurs prior to the remaining carbon compounds. On the other hand, in the burning process at lower temperatures, this second reduction step does not occur.

**Table 2:** Percentage yield of burn processes.

Burn process	Biomass (g)	RHA (g)	R (%)	AGA (g)	R (%)
400 °C/30 min	50.0000	24.7600	49.5200	17.0760	34.1500
800 °C/ 5 h	50.0000	11.1357	22.2700	2.1870	4.3700

### Production of samples

The ashes previously described were grinded and mixed with bakelite (bk) according to three different combinations listed in Table 3. The quantities (in g) of RHA/AGA and bk for preparation of each sample with diameter of 30 mm and thickness of *h* = 4*.*5 mm are also demonstrated in this Table. The solid samples, from these mixtures, were produced using an embedded process. The powdered materials were singly entered in a hydraulic piston and a constant pressure of 1000 lb*/*pol^2^ was applied during a heating ramp from 25 to 145 °C. The final results are pads formed by the aggregation of powdered particles of bk and ashes.

**Table 3:** Concentrations (g) for each bakelite-ash mixture proportions.

Proportions (bk-ashes)	Mass (g)				
	bk	RHA40030	RHA8005h	AGA40030	AGA8005h
100–0% (bk-100)	5.5534	0.0000	0.0000	0.0000	0.0000
60–40%	3.3322	1.2258	1.5920	0.9893	1.8208
50–50%	2.7767	1.7133	1.9890	1.2367	2.5960
40–60%	2.2214	1.9381	2.3870	1.4839	3.1437

**Figure 5:**
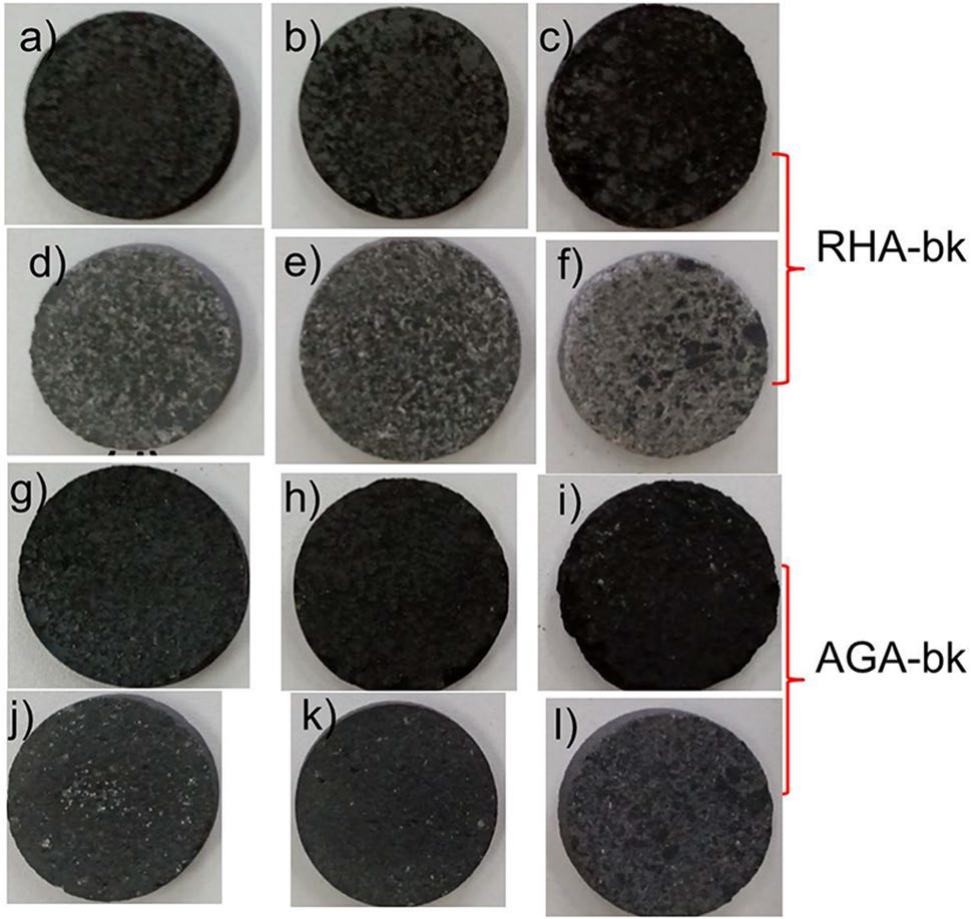
Samples produced from RHA and AGA: (**a**) RHA400301; (**b**) RHA400302; (**c**) RHA400303; (**d**) RHA8005h1; (**e**) RHA8005h2 and (**f**) RHA8005h3; (**g**) AGA400301; (**h**) AGA400302; (**i**) AGA400303; (**j**) AGA8005h1; (**k**) AGA8005h2 and (**l**) AGA8005h3.

Samples produced from RHA-bk and AGA-bk combinations are shown in Fig. 5. The identification codes for RHA-bk combination are: RHA400301 (60% bk–40% RHA40030), RHA400302 (50% bk–50% RHA40030) and RHA400303 (40% bk–60% RHA40030); RHA8005h1 (60% bk–40% RHA8005h), RHA8005h2 (50% bk–50% RHA8005h) and RHA8005h3 (40% bk–60% RHA8005h). Similarly, the identification codes for AGA-bk combinations are: AGA400301 (60% bk–40% AGA40030), AGA400302 (50% bk–50% AGA40030) and AGA400303 (40% bk–60% AGA40030); AGA8005h1 (60% bk–40% AGA8005h), AGA8005h2 (50% bk–50% AGA8005h) and AGA8005h3 (40% bk–60% AGA8005h).

## Results and discussions

### Raman, XRD, SEM, EDS and XRF characterization

**Figure 6:**
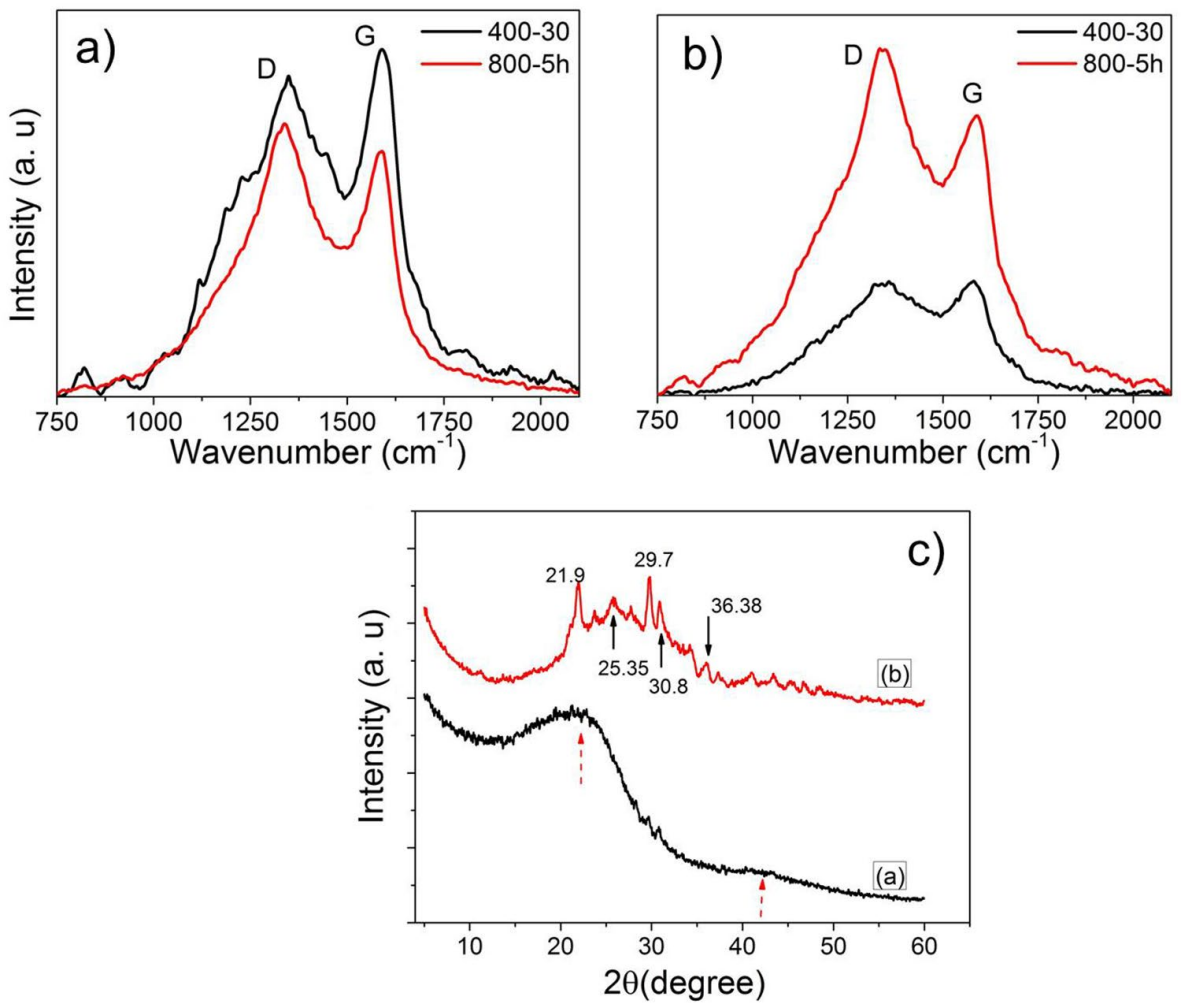
Comparison between the Raman spectra of RHA (**a**) and AG (**b**) burnt at 400 °C/30 min (RHA40030 and AGA40030) and 800 °C/5 h (AGA8005h). (**c**) Comparison between the DRX spectra of AGA40030 (black curve) and AGA8005h (red curve) samples.

Figure 6 shows a comparison between the average Raman spectra (~ 10 Raman spectra taken at different places on the same substrate to compensate for eventual heterogeneity) of RHA (Fig. 6a) and AGA (Fig. 6b) burnt at the temperatures of 400 °C/30 min (RHA40030 and AGA40030) and 800 °C/5 h (RHA8005h and AGA8005h). In Fig. 6a for RHA, it is possible to see that, at 400 °C/30 min the full width half maximum of the D band (FWHM-D) is higher than the FWHM-D at 800 °C/5 h. This fact implies that the disorder is higher on the RHA40030 than on RHA8005h sample, as reported by Severo et al.^25^. Regarding the G band, the intensity is higher on the RHA40030 sample than on the RHA8005h. In this sense, it is well known that, higher intensity of G band than D band contributes greatly to the production of graphitic materials. On the other hand, Fig. 6b shows results for AGA, where it is observed that, at 400 °C/30 min the Raman intensity of the G band (I_G_) is comparable to the Raman intensity of the D band (I_D_), but at 800 °C/5 h, the I_G_ is lower than at 400 °C/30 min, similar to RHA8005h. Accordingly, it is possible to infer that the disorder increases as the burning temperature and time increase, as reported by Severo et al.^25^.

Figure 6c shows a comparison between the XRD measurements of AGA40030 (black curve) and AGA8005h (red curve) samples. For AGA40030, it is possible to observe two wide peaks around 22° and 42° (red arrows), which are compatible with the amorphous structure. This behavior is similar to the XRD patterns of RHA obtained in this work and by other authors at different temperatures. For instance, Muramatsu et al.^26^ have reported two diffraction peaks at 23° and 43° on RHA obtained at 430 °C/2 h; Ghasemi et al.^27^ have shown a main diffraction peak at 21.9° on RHA obtained at 700 °C for 6 h, showing the crystalline phase in the form of cristobalite, tridymite, and quartz. On the other hand, by increasing the temperature and time, the formation of crystalline phases is observed on AGA8005h sample through the appearance of several peaks, some of them shown at 21.9°, 23.5°, 25.8°, 27.7°, 29.7°, 30.8°. This suggests a mixture of phases with different particle sizes. To help to identify the phases of this ash sample, some EDS spectra were acquired in order to verify the constituent elements of this sample (AGA8005h), as well as XRF measurements.

**Figure 7:**
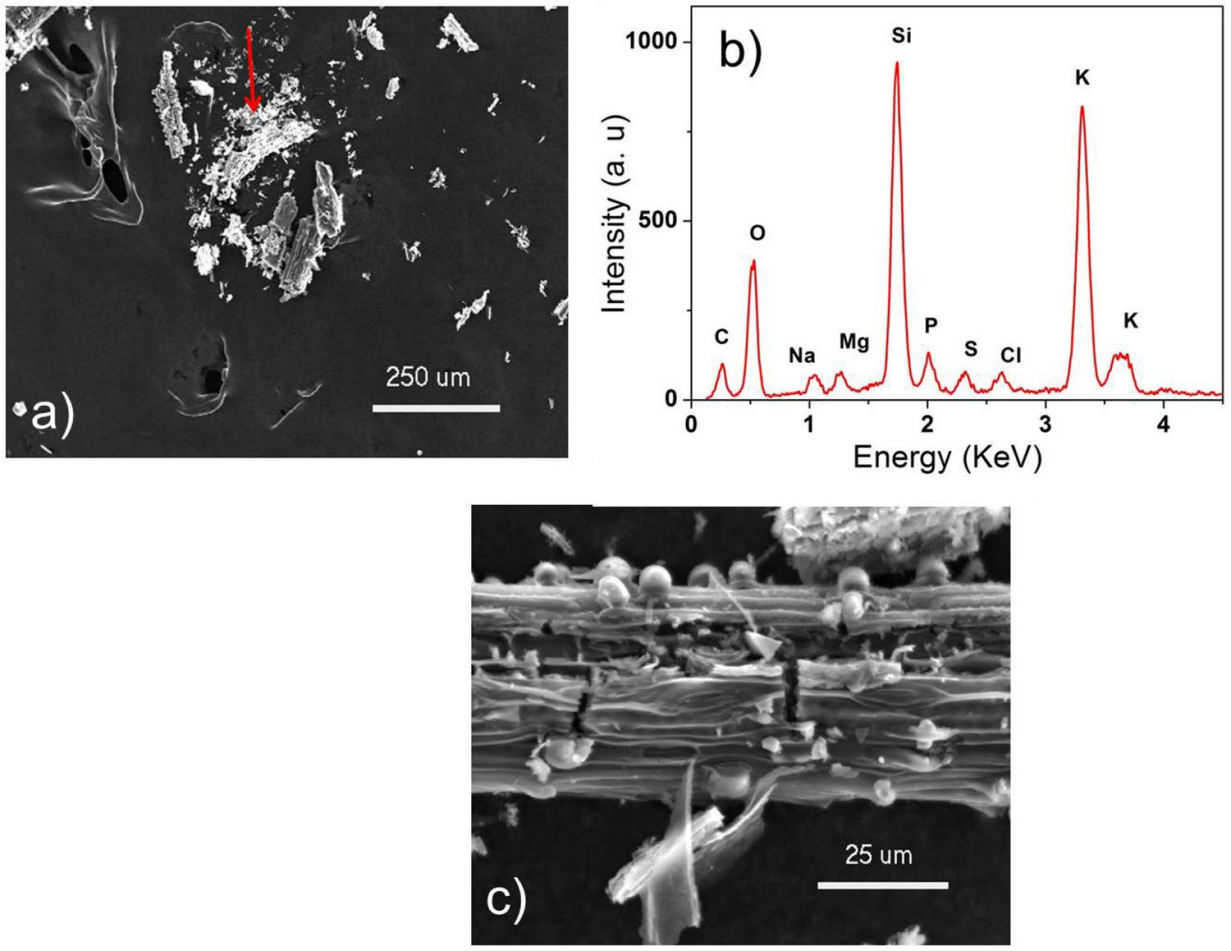
(**a**) SEM image of the AGA8005h powder over a carbon tape; (**b**) EDS spectrum of the region indicated by the red arrow; (**c**) higher magnification of the region indicated by the red arrow, showing the spherulites.

Figure 7a shows a SEM image of the AGA8005h powder over a carbon tape. In the region indicated by an arrow, the EDS spectra in Fig. 7b shows the presence of carbon (C), oxygen (O), sodium (Na), magnesium (Mg), silicon (Si), phosphorus (P), chlorine (Cl), and potassium (K). Higher magnification of this region, in Fig. 7c, shows spherulites deposited over major structures of the powder. EDS spectra acquired at low energy (not shown) in these spherulites, indicates only the presence of Si and O. The spectra from the major structure shows major occurrence of carbon and potassium and minor amounts of the other elements.

From the above observations, it is possible to identify some of the crystalline phases present in the AGA8005h sample. The score for phase identification is higher for cristobalite (PDF 75-0923) in comparison with tridymite or quartz (peaks observed in positions 2θ = 21.9º, 25.35°, 28.4°, 31.45°, and 36.38°). Peaks located at 2θ = 24.0º and 27.7º are compatible with the presence of potassium oxide (PDF 47-1701). Other possible phases present in this sample are more difficult to identify in view of the superposition of crystalline phases and amorphous contribution still present in the difratogram.

Annoni Grass and AGA are unfamiliar bio-materials and its characteristic properties are not yet deeply studied. Therefore the ashes AGA40030 and AGA8005h were also characterized by XRF, in an attempt to verify the estimation of chemical composition of these materials. Table 4 shows the composition, in terms of oxides and chemical reagents and its estimated percentage, of ashes obtained from AG. It is possible to see that SiO_2_, P_2_O_5_, K_2_O, CaO and Al_2_O_3_ are the main compounds on the AGA and their concentrations increase with the temperature.

**Table 4:** Estimated chemical composition of AGA40030 and AGA8005h in XRF analyze.

Compound	AGA40030	AGA8005h
Magnesium oxide—MgO	0.12	3.91
Aluminum oxide—Al_2_O_3_	0.00	12.70
Silicon dioxide—SiO_2_	15.70	40.80
Phosphorus oxide—P_2_O_5_	1.46	6.52
Sulfur—S	0.59	1.67
Chlorine—Cl	0.61	0.98
Potassium oxide—K_2_O	12.40	16.00
Calcium oxide—CaO	5.37	7.36
Iron oxide—Fe_2_O_3_	0.45	2.02

### Electromagnetic characterization

**Figure 8:**
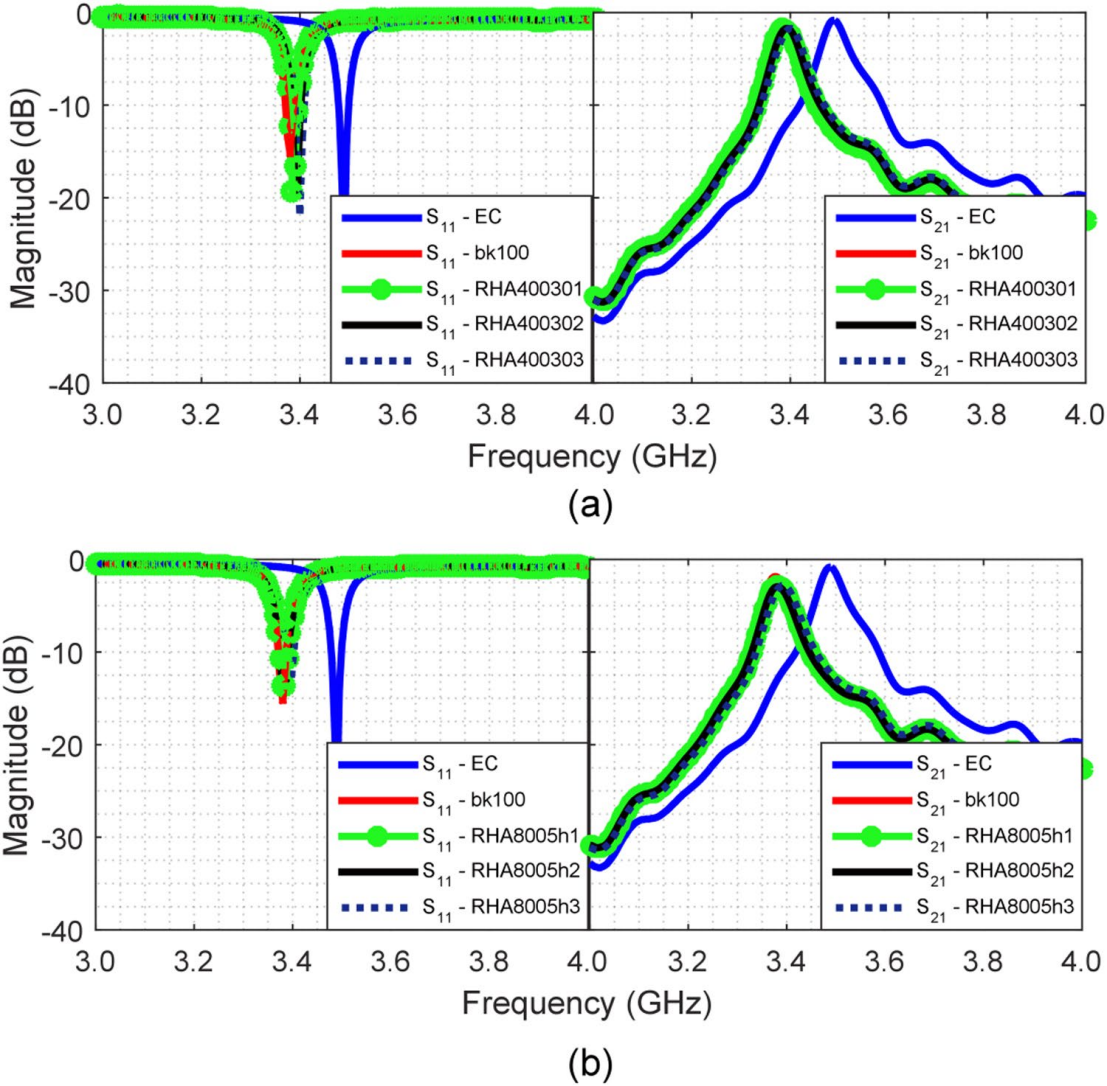
Measured results: (**a**) *S*_11_ and *S*_21_ parameters for the RHA40030 sample; (**b**) *S*_11_ and *S*_21_ parameters for the RHA8005h sample.

The measured *S*_11_ and *S*_21_ parameters for the characterization of RHA40030/bk and RHA8005h/bk samples (shown Fig. 5a–f) are demonstrated in Fig. 8a,b, respectively. These results are compared with the measured curves for the empty cavity (blue curve) and the reference sample (bk100, red curve). The corresponding value of ${f}_{a}$ for each sample was inserted in Eq. (3) for the respective calculation of the ${\varepsilon }_{r}$ constants. The loss tangent $\mathrm{tan \delta }$ was obtained from *S*_21_ ${(f}_{a})$ (magnitude of the *S*_21_ parameter at ${f}_{a}$), by means of parametric simulations in HFSS®. These parameters are listed in Table 5.

**Table 5:** Parameters of characterization of the dielectric properties of RHA-BK samples.

Sample	${f}_{a}$ (GHz)	*S*_21_ $({f}_{a})$ (dB)	${\varepsilon }_{r}$	$tan\,\delta$
bk-100	3.3750	− 1.8390	5.89	0.0840
RHA400301	3.3875	− 1.7500	4.51	0.0620
RHA400302	3.3925	− 1.7090	4.07	0.0600
RHA400303	3.4000	− 1.6960	3.52	0.0575
RHA8005h1	3.3800	− 2.7880	5.28	0.1480
RHA8005h2	3.3825	− 2.9300	5.01	0.1690
RHA8005h3	3.3950	− 2.8170	3.87	0.1650

Samples produced from RHA40030 and bk have dielectric constants varying between 4.51 (RHA400301) and 3.52 (RHA400303), and dielectric-loss tangent with values between 0.0620 and 0.0575, respectively. The values of these constants were controlled according to the ash concentration (%) in the mixture, because in this case, the addition of high quantity of this material causes the reduction of ${\varepsilon }_{r}$ and $\mathrm{tan \delta }$ of the samples, whilst the reference sample bk100 presented ${\varepsilon }_{r}$ = 5.89 and $tan \delta$ = 0.0840. Thereby, it can be inferred that RHA40030 has ${\varepsilon }_{r}$ and $\mathrm{tan \delta }$ constants lower than those ones of bk, and can be also lower than 3.52 and 0.0575, respectively. It is worth to emphasize that Aleem et al.^28^ reported the standard dielectric constant value of Bakelite around 4.50–5.50, for a frequency range of 0.2–3.0 GHz. But, Buyukozturk et al.^29^ reported a dielectric constant value of 5.95 for a frequency range of 8.0–18 GHz. In spite of, the frequency range of these authors be different from the frequency used in this work (3.49 GHz), they have used the transmission coefficient and time difference of arrival (TDOA) method, and not the resonant cavity method. Additionally, they also reported different values for the dielectric constant of Bakelite, when different ranges of frequencies are used for its measurement. Then, according to this, we can argue that the value of ${\varepsilon }_{r}$ = 5.89 for Bakelite found in this work, using the resonant cavity method is within an acceptable level.

**Figure 9:**
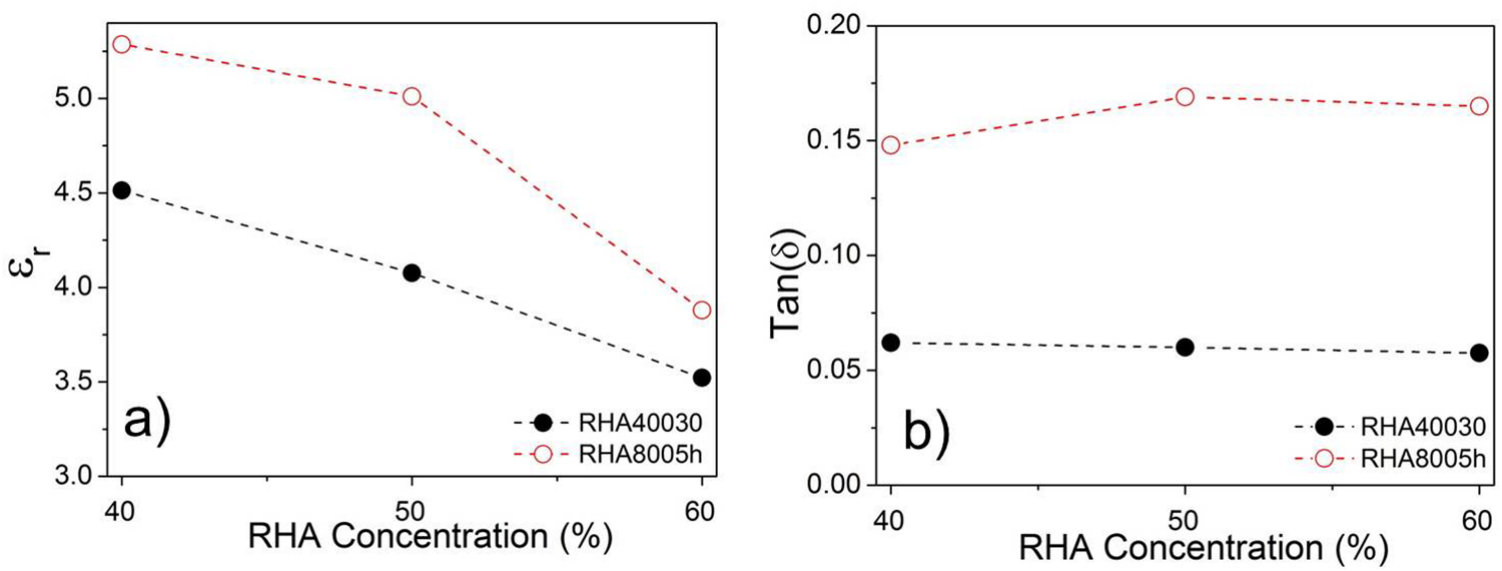
Variation of the properties according to RHA concentrations: (**a**) ${\varepsilon }_{r}$; (**b**) $\mathrm{tan\,\delta }$.

On the other hand, the calculated ${\varepsilon }_{r}$ and estimated $\mathrm{tan \delta }$ constants for samples produced from RHA8005h/bk mixtures were higher than those ones obtained for RHA40030/bk. This fact can be explained because RHA8005h is denser than RHA40030 and has higher concentration of silica in its composition, which is in agreement to the value ${\varepsilon }_{r}$ = 3.90 reported by Robertson^30^. Table 5 shows that, the dielectric constant and dielectric-loss tangent of these samples varied in the range 5.28–3.87, and 0.1480–0.1690, respectively. Graphically, the variation of properties of ${\varepsilon }_{r}$ and $\mathrm{tan \delta }$ of the samples RHA40030/bk (filled circles) and RHA8005h/bk (open circles) are presented in Fig. 9, respectively. The inverse behavior between ${\varepsilon }_{r}$ and RHA concentration (%) was observed for the two sample groups. The variation of $\mathrm{tan \delta }$ demonstrates that RHA40030 has lower dielectric losses in comparison to RHA8005h.

As reported before, Liu et al.^7^ reported a value of ${\varepsilon }_{r}$~ 3.00 for RHA burnt at the temperature of 800 °C, which is lower than the value found in this work for the same temperature (3.87- 5.28). This difference is probably due to the burning time and the frequency range used by them, which are of 2 h and from 2 to 10 GHz. Garnayak et al.^5^, however, reported the experimental characterization of samples based from a mixture of RHA (30 wt%), obtained from two stages of burning processes (400 °C—5 h and 600 °C in sequence), and epoxy resin (70wt%). The frequency range for this analysis was 8.2–12.4 GHz (X- band). The dielectric constant and dielectric-loss tangent of their samples presented values varying between 4.48–4.29, and 0.0630–0.0800, respectively. In spite of, the authors used epoxy resin, and not Bakelite, as a binding agent and a RHA concentration of only 30wt %, as well as, different frequency ranges, their experimental results are similar to those obtained in this work. Also, in the present study, samples composed with 60wt% RHA were characterized and the obtained ${\varepsilon }_{r}$ and $\mathrm{tan \delta }$ properties are predominantly influenced by the ashes instead of by the binding material (bk), then these results are very similar to the dielectric properties of pure ashes.

It is worth to emphasize that, several authors have reported different dielectric constant values for similar materials with similar preparation. In this sense, according to Seng et al.^3^ the dielectric constant value of some materials depend of the frequency ranges, where the measurements are performed. Besides, the methodology used to be the measurements, thickness of samples have also influence. These are probably the reasons, why it is possible to find on the literature, different dielectric constant values for similar materials with similar preparation.

In addition, Lee et al.^31^ and Nath et al.^32^ used RHA in mixtures with polymeric resins for the production of electromagnetic absorbing composite material for applications in stealth technology. This possibility of use of RHA in Communications is pertinent, because, as related in the literature and in present study, composites produced from RHA have high $\mathrm{tan \delta }$ constant, hence resulting in a good capacity of absorption of electromagnetic energy. Additionally, as it is a reusable material from abundant biomass and, consequently, very easy to obtain, it is quite competitive and interesting to be used in the development of radiation absorbent materials (RAM). In addition, low dielectric-loss tangent was also reported by other authors^28,33^ on glasses fabricated using RHA as raw material. In this sense, this study open the possibility of using the RH, and consequently RHA, produced on Brazilian territory as source for developing microwave absorption materials.

**Figure 10:**
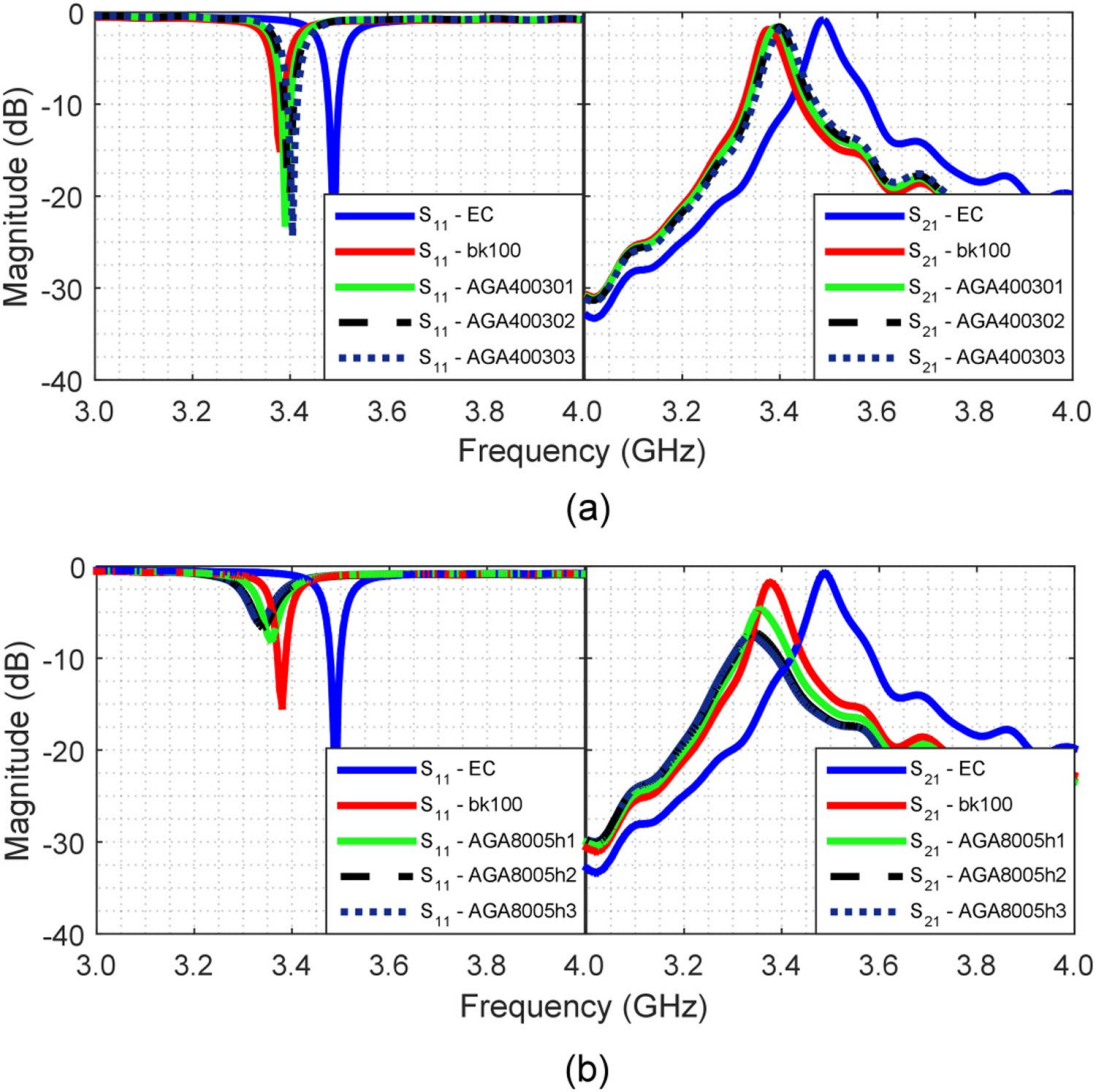
Measured results: (**a**) *S*_11_ and *S*_21_-parameters for AGA40030 sample; and (**b**) *S*_11_ and *S*_21_-parameters for AGA8005h sample.

Similarly, samples produced from AGA/bk mixture (shown Fig. 5g–l) were also characterized according to the previously described methodology. Measured *S*_11_ and *S*_21_ parameters for samples AGA40030/bk and AGA8005h/bk are demonstrated in Fig. 10a,b, respectively. From *S*_21_(${f}_{a}$) the constants ${\varepsilon }_{r}$ and $\mathrm{tan \delta }$ were calculated and are listed in Table 6.

**Table 6:** Parameters of characterization of the dielectric properties of AGA-BK samples.

Sample	${f}_{a}$ (GHz)	*S*_21_(${f}_{a}$) (dB)	${\varepsilon }_{r}$	$tan\,\delta$
bk-100	3.3750	− 1.8390	5.89	0.0840
AGA400301	3.3900	− 1.6120	4.28	0.0525
AGA400302	3.3975	− 1.5580	3.69	0.0490
AGA400303	3.4050	− 1.6190	3.20	0.0528
AGA8005h1	3.3550	− 4.7310	9.10	0.4540
AGA8005h2	3.3000	− 7.1950	11.84	1.2500
AGA8005h3	3.3600	− 7.5530	15.14	1.4900

**Figure 11:**
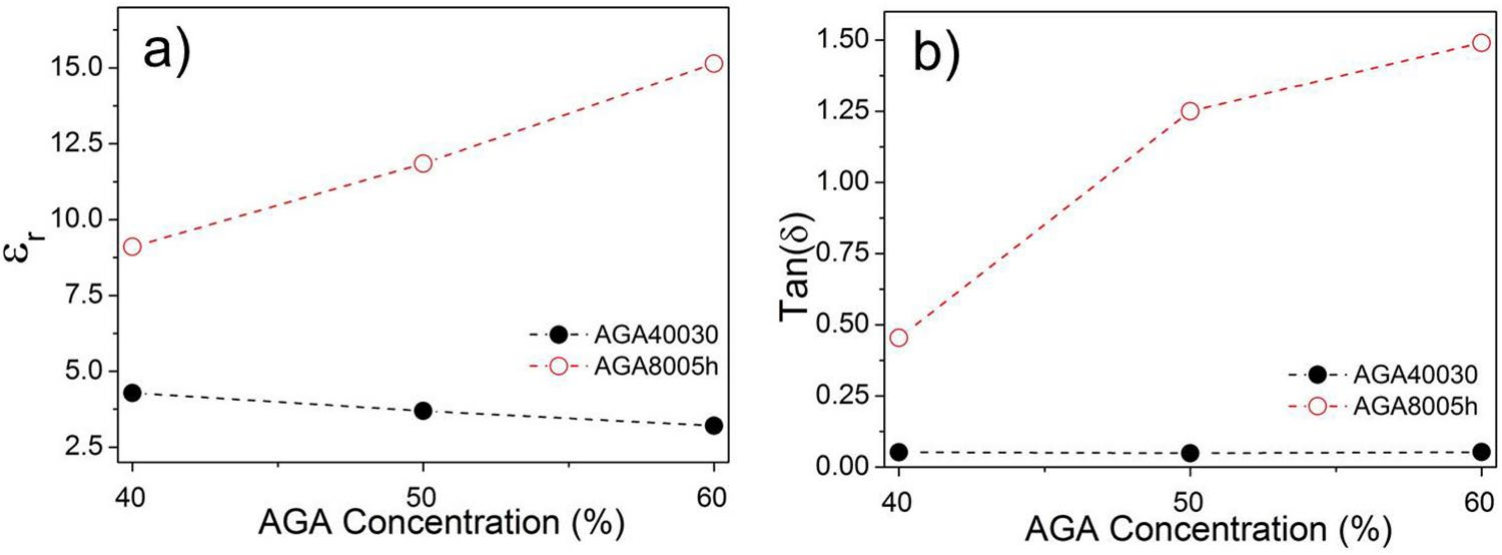
Variation of properties according AGA concentrations: (**a**) ${\varepsilon }_{r}$; and (**b**) $\mathrm{tan\,\delta }$.

Samples based in AGA40030/bk mixtures presented lower dielectric constants, varying between 4.28 and 3.20, hence indicating that is inversely related to the concentration of ashes (filled circles in Fig. 11a). This behavior was also verified previously in the characterization of the dielectric properties of samples RHA40030/bk and indicates that this material exhibits dielectric constant lower than 3.20. On the other hand, the dielectric-loss tangent for these samples, shown as filled circles in Fig. 11b, presented very small variation with the increase of ash concentration in the mixture, whereby the difference between the smallest and largest value of $\mathrm{tan \delta }$ is ca. 0.0038.

Samples produced from AGA8005h/bk mixtures, however, presented very high values of both relative permittivity and loss tangent. The characterized ${\varepsilon }_{r}$ constants significantly increase with the increase of AGA8005h concentration, varying between 9.10 and 15.14 (open circles in Fig. 11a). This same behavior was verified for $\mathrm{tan \delta }$ constant that presented variation between 0.4540 and 1.4900 (open circles on Fig. 11b). These values, especially those of dielectric-loss tangent, are considered larger than those obtained for the other samples produced from RHA40030, RHA8005h and AGA40030 and indicate that the obtained AGA8005h has formidable features for RAM manufacturing.

According to the data listed in Table 4 and EDS results, AGA is formed by different oxides and chemical elements, with different concentrations (%). Specifically to AGA8005h, the predominant reagents are: silicon dioxide (40.80%), potassium oxide (16.00%), aluminum oxide (12.70%), calcium oxide (7.36%) and phosphorus oxide (6.52%). One can argue that the combination of these oxides, which increases with the temperature, and also the presence of disordered carbon molecules (as shown in Raman measurements) results in ashes with very high dielectric-loss tangent and, consequently, extreme high capability of absorption of electromagnetic radiation.

The sample AGA8005h is the bio-material that presented best performance for electromagnetic energy absorption, according to characterized dielectric properties previously discussed. This residue is obtained from an invasive grass species existing in Southern Brazil, whose removal from nature is very important to preservation of native grass species. For these reasons, it is worth to develop studies about the production of new composite materials based on the combination of AGA8005h and other ashes, such as RHA40030 and RHA8005h, whose yields are larger and their precursor biomass is also abundantly found in the region.

## Conclusions

In this work, the characterization of dielectric properties of samples of four different ashes of rice husk and annoni grass at 3.5 GHz, which is the test frequency of the 5th generation of mobile communication systems (5G) was presented. Each sample was produced according to a mixture of specified concentrations (%) of ashes and bk. The experimental setup for the measurements was developed using the resonant cavity method. The larger results in terms of constants were obtained for RHA8005h/bk and AGA8005h/bk samples, highlighting that the results for AGA8005h/bk are extremely higher than those for the other materials. According to XRF and EDS measurements, this phenomenon is due to its chemical composition based on different inorganic oxides, including SiO_2_ (40.80%), K_2_O (16.00%), Al_2_O_3_ (12.70%), CaO (7.36%) and P_2_O_5_ (6.52%) as the predominant ones. These results and the possibility of controlling the material properties by varying the concentration of AGA8005h or RHA8005h in a specified matrix is very interesting for the development of new composites with very good capacity of electromagnetic energy absorption.

## Data Availability

All data generated or analysed during this study are included in this published article.
